# Dynamic localization of DNA topoisomerase I and its functional relevance during Drosophila development

**DOI:** 10.1093/g3journal/jkab202

**Published:** 2021-06-14

**Authors:** Wuqiang Huang, Zhiping Liu, Yikang S Rong

**Affiliations:** 1 School of Life Sciences, Sun Yat-sen University, Guangzhou, Guangdong Province, 510275, China; 2 Hengyang College of Medicine, University of South China, Hengyang 421001, China

**Keywords:** topoisomerase I, rDNA and nucleolus, meiotic chromosome segregation, histone locus body

## Abstract

DNA topoisomerase I (Top1) maintains chromatin conformation during transcription. While Top1 is not essential in simple eukaryotic organisms such as yeast, it is required for the development of multicellular organisms. In fact, tissue and cell-type-specific functions of Top1 have been suggested in the fruit fly Drosophila. A better understanding of Top1’s function in the context of development is important as Top1 inhibitors are among the most widely used anticancer drugs. As a step toward such a better understanding, we studied its localization in live cells of Drosophila. Consistent with prior results, Top1 is highly enriched at the nucleolus in transcriptionally active polyploid cells, and this enrichment responds to perturbation of transcription. In diploid cells, we uncovered evidence for Top1 foci formation at genomic regions not limited to the active *rDNA* locus, suggestive of novel regulation of Top1 recruitment. In the male germline, Top1 is highly enriched at the paired *rDNA* loci on sex chromosomes suggesting that it might participate in regulating their segregation during meiosis. Results from RNAi-mediated Top1 knockdown lend support to this hypothesis. Our study has provided one of the most comprehensive descriptions of Top1 localization during animal development.

## Introduction

DNA topoisomerase I (Top1) in eukaryotic organisms belongs to type I topoisomerases that produce a transient single-stranded DNA break to relieve torsion generated during transcription and other processes (Wang 1985; Pommier *et al.* 1998). Top1 inhibitors have been widely used in cancer therapies, most of them cause cytotoxicity by trapping the cross-linked DNA-Top1 intermediate, thus interfering with processes such as DNA replication and transcription (Thomas and Pommier 2019). Therefore, a better understanding of Top1’s *in vivo* function carries medical significance. 

Loss of Top1 function is tolerated in yeast with elevated instability at the *rDNA* locus (Christman *et al.* 1988; Andersen *et al.* 2015), perhaps the most active locus in the genome, suggesting that Top1 is needed to prevent the accumulation of recombinogenic intermediates at *rDNA* during transcription. How Top1 is recruited to transcriptionally active loci has been extensively studied in many different organisms. At the nucleolus, where rDNA transcription by RNA polymerase I (Pol I) happens, Top1’s accumulation depends on its ability to interact with Pol I (Rose *et al.* 1988; Christensen *et al.* 2004). At other active loci such as the heat shock genes in Drosophila, Top1’s recruitment might involve interaction with RNA Pol II or directly with the transcribed region (Gilmour *et al.* 1986; Gilmour and Elgin 1987; Shaiu and Hsieh 1998). In addition to interactions with the transcriptional machineries, other mechanisms of Top1’s recruitment have been implicated, such as interacting with supercoiled DNA (Muller 1985; Zechiedrich and Osheroff 1990) or with specific DNA sequences (Bonven *et al.* 1985; Christiansen *et al.* 1987). Therefore, our understanding of the enzymology and of the recruitment of Top1 by transcriptional activities is extensive, yet that of Top1’s *in vivo* functions, particularly those in the developmental context or independent of transcription, is less so in comparison.

In contrast to simpler eukaryotes, loss of Top1 does not support life in complex organisms such as the fruit fly Drosophila (Lee *et al.* 1993) or the worm *C*aenorhabditis *elegans* (Lee *et al.* 2014). Several functions of Top1 that might be specific to higher eukaryotes have been shown or proposed in different organisms. In early embryos of Drosophila, in the absence of ongoing transcription genome wide, a reduction of Top1 level leads to mitotic catastrophe (Zhang *et al.* 2000). A fly *Top1* mutation suppresses seizure by regulating neuronal cell death (Song *et al.* 2007). Top1’s function in the meiotic segregation of nonexchanged chromosomes in Drosophila has been proposed (McKee *et al.* 1992), and its activity is weakly required for pairing of homologous chromosomes in mouse meiosis (Cobb *et al.* 1997). In addition, Top1 purified from human and Drosophila cells possesses kinase activities toward some of the SR proteins that participate in splicing regulation (Rossi *et al.* 1996; Juge *et al.* 2010), suggesting an additional mode of Top1 action in the regulation of gene expression.

A better understanding of Top1 in complex organisms may require a thorough description of its localization in different tissue and cell types. Previously, studies of Top1 localization in Drosophila cells have been limited to polytene cells from larval salivary glands (Gilmour and Elgin 1987; Shaiu and Hsieh 1998; Zobeck *et al.* 2010) and cells in the earliest divisions (Zhang *et al.* 2000). Here we conducted a more thorough investigation into the cellular localization of Top1 in developmental stage and cell-type-specific manners, focusing primarily on interphase cells. We uncovered new features of Top1 distribution that may have important implications for its function.

## Materials and methods

### Drosophila stocks

The *Top1* mutation was generated as described below. The *Top1^40^* and *Top1^26^* alleles were used in this study. The *Top1^40^* and *Top1^26^* alleles carry an 8-bp and a 1-bp deletion of the *Top1* coding region, respectively, both leading to a frame-shift mutation with prematured stop codons downstream of the mutation. A stock with the *Top1^CC01414^* allele and one with a marked *Y* chromosome (*B^S^Yy^+^*) were obtained from the Bloomington Stock Center. Fly lines carrying the *bam-gal4* and *nos-gal4* drivers were gifts from Prof. Xin Chen at Johns Hopkins University, and *topi-gal4* was a gift from Prof. Christian Lehner at the University of Zurich.

### Cas-9-mediated mutagenesis of *Top1*

We generated new *Top1* alleles with CRISPR-Cas9-mediated mutagenesis using a transgenic approach in which both the Cas9 protein (expressed from a *vasa* promoter) and gRNA (expressed from a *U6* promoter) were produced from transgenes inserted into the Drosophila genome (Port *et al.* 2014). The target gRNA was designed with an online tool: http://tools.flycrispr.molbio.wisc.edu/targetFinder/. A target sequence was chosen that has the sequence of 5′-GGCGCGCAAGAAGGTTAAGAAGG with the PAM sequence in bold. Mutations were verified by genomic PCR and sequencing using DNA samples from homozygous mutant larvae.

### Construction of *Top1-gfp* and *Top1^Y932F^-gfp* transgenic lines

DNA from the *Top1* locus is carried by the BAC Clone #48D17 and was subcloned into the pUAST-attB (Bischof *et al.* 2007) vector by gap repair based on the technique of “recombineering” (Venken *et al.* 2006; Wesolowska and Rong 2013), and the *gfp* gene was subsequently inserted at the end of Top1 coding region before the stop codon by recombineering (Zhang *et al.* 2014). *Top1-gfp* fly line was generated by PhiC31 integrase-mediated germline insertions on chromosomes *II* (25C) and chromosome *III* (86F). To test the ability of *Top1-gfp* to rescue the lethality of *Top1* mutant animals, female flies heterozygous for the *X*-linked *Top1* frameshift mutations were mated to wild-type males carrying a single copy of the *Top1-gfp* transgene inserted on an autosome. Male progeny that carried the *Top1* mutation and *Top1-gfp* survive while those carrying *Top1* mutation alone were not recovered. For progeny counts from this rescuing cross, see Supplementary Table S1 in Supplemental Materials. Both chromosome *II* and *III* insertions of *Top1-gfp* rescued the lethality. The insertion on chromosome *II* was used primarily for localization studies.

### Live GFP imaging

For live imaging, wing discs and salivary glands from third instar larvae, and testes and ovaries from adult flies were dissected in PBS, placed on a coverslip in a drop of PBS, squashed gently by the weight of the slide lowered on top before imaging.

### Immunostaining

Embryo collections were performed on grape juice plates for 2 h, dechorionated with 50% bleach and washed with embryo wash buffer (0.7% NaCl, 0.05% TritonX-100), fixed with a 1:1 mixture of freshly diluted 3.7% formaldehyde in PBS and heptane for 30 min, and devitellinized in methanol and heptane (1:1) following the slow formaldehyde fix method described by Sullivan *et al.* (2000). Purified TALE-mCherry proteins were kindly provided by Prof. Kai Yuan of the Central South University in China and used as an antibody in localizing the 359 satellite. A mouse MPM-2 antibody from Abcam was used at 1:1000.

Larval or adult tissues were dissected in fresh PBS, fixed with a 1:1 mixture of freshly diluted 3.7% formaldehyde in PBS and heptane for 30 min at room temperature. Tissues were then washed three times in PBST (1× PBS containing 0.1% of Tween 20) for 15 min each, blocked in 3% bovine serum albumin (BSA) in PBST for 1 h at room temperature, followed by an overnight incubation with a rabbit anti-Fib (1:400, Abcam) in 3% BSA in PBST at 4°C. After three washes as above, the samples were incubated in Alexa Fluor 555 conjugated goat antirabbit IgG (1:200, Thermal) for 1 h at room temperature, followed by three washes in PBST and stained with DAPI for 10 min, and mounted in VECTASHIELD.

Confocal microscopy was performed on an Olympus FV1200 (equipped with VIS, UV, and IR lasers). Images were processed with photoshop and Illustrator (CS6; Adobe).

### Actinomycin D and heat shock treatments of larval salivary glands

Salivary glands were dissected from third instar larvae in 1× PBS and then incubated with 0.05, 0.5, 1, or 10 µg/ml of actinomycin D (ActD) solution for 1.5 h at room temperature. Heat shock treatment of salivary glands was done by treating the glands in Schneider’s Drosophila Medium for 30 min at 37°C in a water bath. Finally, the salivary glands were stained with 2 µg/ml of Hoechst 33342 for 10 min. Fluorescence observation was carried out under a Zeiss microscope with a 40× objective.

### Data availability

Strains and plasmids are available upon request. The authors affirm that all data necessary for confirming the conclusions of the article are present within the article, figures, and tables.

Supplementary material is available at *G3* online.

## Results and discussion

### A *gfp*-tagged *Top1* transgene for the study of Top1 localization

**Figure 1:**
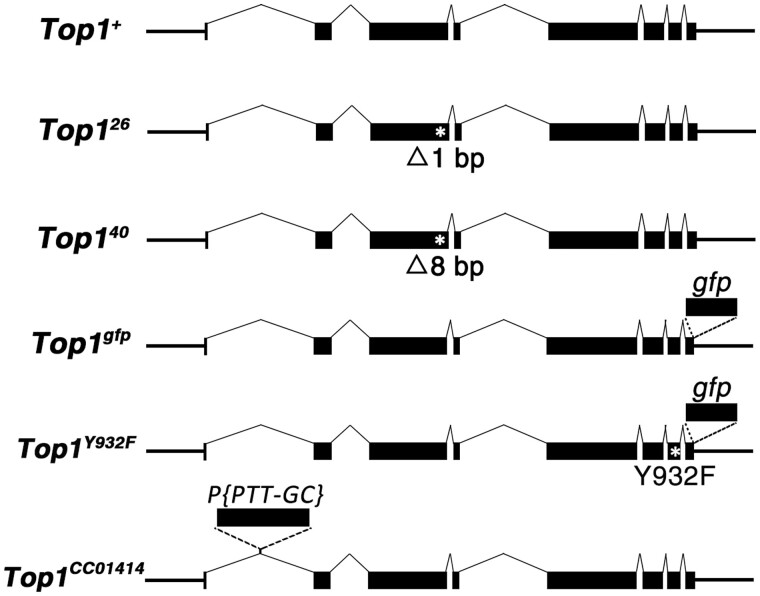
*Top1* alleles used in the study. The *wt Top1* locus is shown at the top with exons denoted as black boxes, which are connected with lines that indicate introns. The two point mutations (*Top1^26^* and *Top1^40^*) are shown below with the approximate position of the small deletions indicated with an asterisk. The approximate position of the insertional site of the GFP tag is indicated for *Top1^gfp^*. In the *Top1^Y932F^* allele, the Y to F change is denoted by an asterisk and the insertion of *gfp* at the C-terminus is also indicated. The *Top1^CC01414^* allele has been described in Morin *et al.* (2001) and FlyBase, and the approximate position of the insertion site of the *P* element is indicated.

The best approach for tagging a protein with GFP is to knock-in a *gfp* fragment to the endogenous locus. Unfortunately, our repeated attempts at the *Top1* locus turned out unsuccessful. To monitor Top1 protein localization, we constructed a transgene carrying the endogenous regulatory elements of the *Top1* locus and expresses Top1 protein fused with GFP at its C-terminus (Figure 1). To clone the relatively large size of the *Top1* locus, we used the method of gap repair by recombineering (Venken *et al.* 2006; Wesolowska and Rong 2013) and successfully inserted a 13-kb genomic fragment of the *Top1* locus with about 1 kb each of upstream and downstream sequences (Figure 1). Also using the recombineering method, we inserted the coding sequence for GFP just upstream of the stop codon of *Top1* (Zhang *et al.* 2014).

To test the suitability of this tagged *Top1* transgene in reflecting the *in vivo* behavior of the endogenous untagged *Top1*, we introduced it into *Top1* frameshift mutations that we generated by CRISPR-mediated mutagenesis (see *Materials and Methods*) and discovered that it rescued the lethality caused by the mutations so that flies carrying both the *Top1* mutation and a single copy of the *Top1-gfp* transgene on an autosome were recovered normally (Supplementary Table S1). This suggests that our Top1-GFP likely assumes functional localization similar to the endogenous Top1 proteins. To assess the influence of Top1 enzymatic activity on its cellular localization, we constructed an identically tagged *Top1* transgene with the Tyrosine residue of the active site mutated to a Phenylalanine residue (the *Top1^Y932F^* mutation). As expected, this mutated form of Top1 was not able to rescue the lethality caused by *Top1* frameshift mutations. Nevertheless, under an otherwise wild-type background, it displays nuclear GFP signals similar to that of Top1-GFP (see later sections).

A *gfp*-tagged *Top1* allele (*Top1^CC01414^*) has been recovered previously in a transposon-mediated gene tagging project (Morin *et al.* 2001). In this allele, a *P* element was inserted in the first intron of *Top1*. A pre-mRNA splicing reaction using the splicing donor-acceptor sites in the *P* element results in an in-frame fusion of *gfp* with the rest of the *Top1* coding region. Indeed, this allele has been used previously to monitor Top1 localization using live GFP fluorescence in larval polytene cells (Zobeck *et al.* 2010). Our *gfp*-tagged *Top1* transgene potentially has given us two modest advantages over the *P* element tagged allele. First, it simplified our introduction of the active site mutation (*Top1^Y932F^*) and other *Top1* mutations in the future. Secondly, since GFP-tagged Top1 protein expressed from the *Top1^CC01414^* allele has to be produced by an ectopic splicing reaction, alternative splicing using the endogenous splicing sites of exon 1 would generate Top1 proteins lacking GFP. Indeed, multiple *Top1* isoforms are predicted by genomic annotation (flybase.net) even though molecular analyses of *Top1* transcripts have yet to lend support for their existence (Brown *et al.* 1998). Nevertheless, as shown in later sections, whenever we compared localization patterns of GFP-tagged Top1 produced from either experimental set up (transgene *vs* “tagging by splicing”), we obtained very similar if not identical results. This series of “control” experiments further validated our transgenic approach for studying Top1 localization.

### Nucleolar enrichment of Top1 in metabolically active polyploid cells

**Figure 2:**
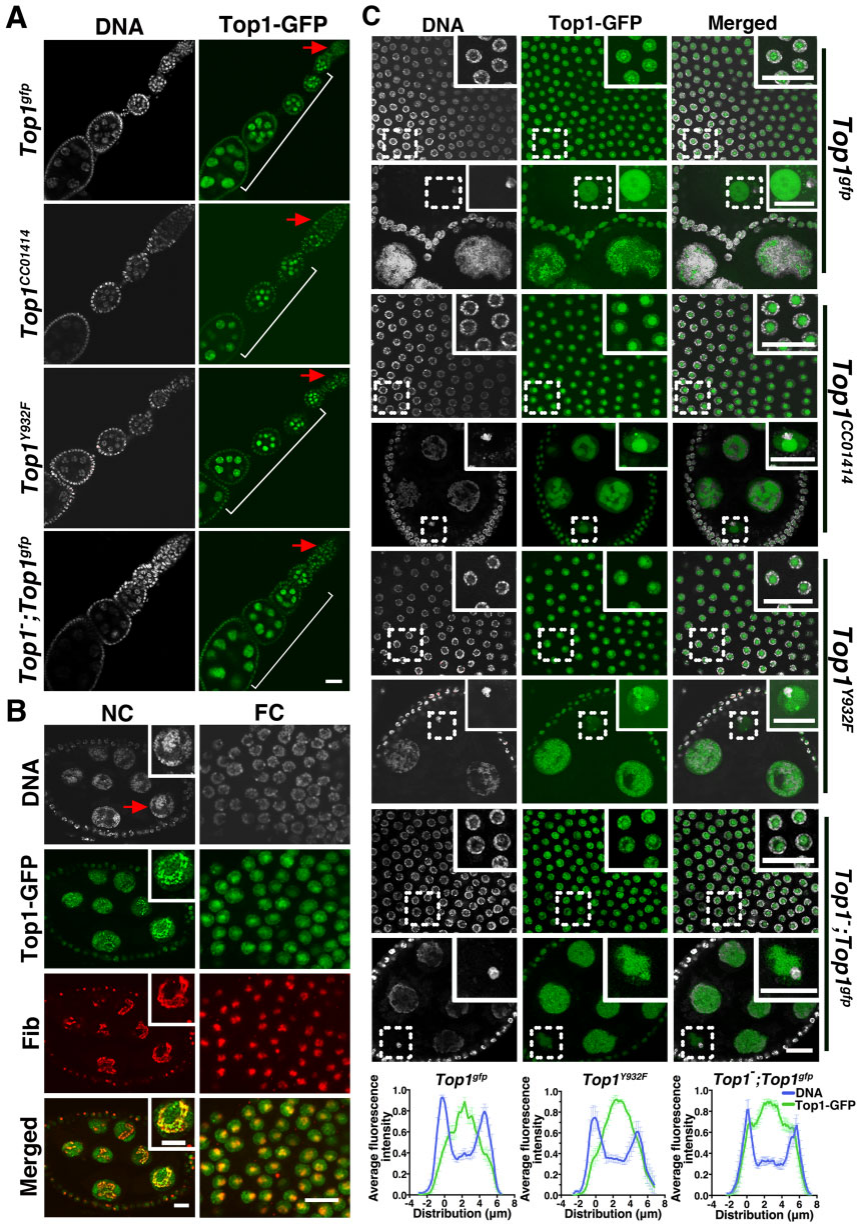
Top1-GFP localization in the female germline. (A) Overview of Top1-GFP localization in the ovary. A single ovariole containing the germarium (arrow), early- and mid-staged egg chambers (bracket) is shown for four genotypes (shown to the left of the pictures). Nuclear GFP signals are abundant in the large nurse cells, which are surrounded by follicle cells that also display GFP fluorescence. Scale bar represents 20 µm. (B) Top1-GFP colocalization with Fib, a nucleolar marker. Fluorescence from Top1-GFP colocalizes with anti-Fib signals (red) in both the larger nurse cells (NC, also indicated with an arrow) and the smaller follicle cells (FC). The “Merged” image shows signals from Top1-GFP and Fib antibody. Magnified images of the nurse cell marked with the arrow are also provided as inserts. Scale bars represent 10 µm. (C) Top1-GFP localization in germlines with four different genotypes. Genotypes are listed to the right of the pictures. For each genotype, GFP signals in follicle cells (top panels), nurse cells and the oocyte (lower panels) are shown. Magnified images of the areas marked with dashed rectangles are shown as inserts. The nuclear genome of the oocyte is DAPI-bright and often juxtaposes or overlaps with a large Top1-GFP sphere. Scale bar represents 20 µm. Line-scan analyses from eight follicle cells for each genotype are provided at the bottom, using a method described in Billmyre *et al.* (2019). Error bars indicate SEM.

One of the most important function of Top1 is in transcription regulation, particularly in the regulation of rRNA synthesis by RNA Pol I. Consistently, Top1 is enriched in the nucleolus of eukaryotic cells (Fleischmann *et al.* 1984; Muller *et al.* 1985; Zhang *et al.* 2000; Christensen *et al.* 2004; Juge *et al.* 2010; Cha *et al.* 2012). We investigated whether Top1 is similarly distributed in metabolically active but nondividing cells in the female germline and observed nuclear GFP signal in ovaries of flies carrying the *Top1^gfp^*, *Top1^Y932F^*, or *Top1^CC01414^* alleles individually (Figure 2). More specifically, Top1 can be seen on chromatin in polyploid nurse cells but with a prominent enrichment at a “chromatin-poor” region that likely corresponds to the nucleolus. In somatic follicle cells, which are also polyploid in later egg chambers, Top1 has a similar bipartite mode of distribution: weaker GFP fluorescence on chromatin and a strong enrichment in the presumed nucleolus (Figure 2, B and C). To confirm the region with the strongest Top1-GFP signal does represent the nucleolus, we used Fibrillarin (Fib) as a nucleolar marker in antibody staining of ovaries expressing Top1-GFP and observed colocalization of GFP and anti-Fib signals (Figure 2B). Interestingly, Top1-GFP localization in the nucleolus is not uniform but displays very strong puncta (Figure 2B) that might represent the “Fibrillar Centers” previously identified as Top1-rich (Muller *et al.* 1985; Christensen *et al.* 2004). To investigate whether the overproduction of Top1 proteins, because of the presence of the *gfp*-tagged *Top1* transgene, might have led to abnormal localization of Top1-GFP, we studied its localization in the female germlines that are also homozygous for the *Top1* frameshift mutation on the *X* chromosome. The distribution pattern of Top1-GFP in these “rescued” germlines is indistinguishable from that in the “over-expressed” germlines (Figure 2, A and B).

Besides somatic follicle cells and germline nurse cells, which are metabolically active to support oogenesis, we also observed Top1-GFP in the nucleus of the oocyte (Figure 2C). Interestingly, Top1-GFP forms a large body inside the oocyte nucleus that is separated from the bulk of chromatin. A large “extra-chromatin” body inside the oocyte was previously observed for Top1 (Liu *et al.* 2006a) and Nopp140, another nucleolar protein (McCain *et al.* 2006). The biological function of this large structure remains obscure.

**Figure 3:**
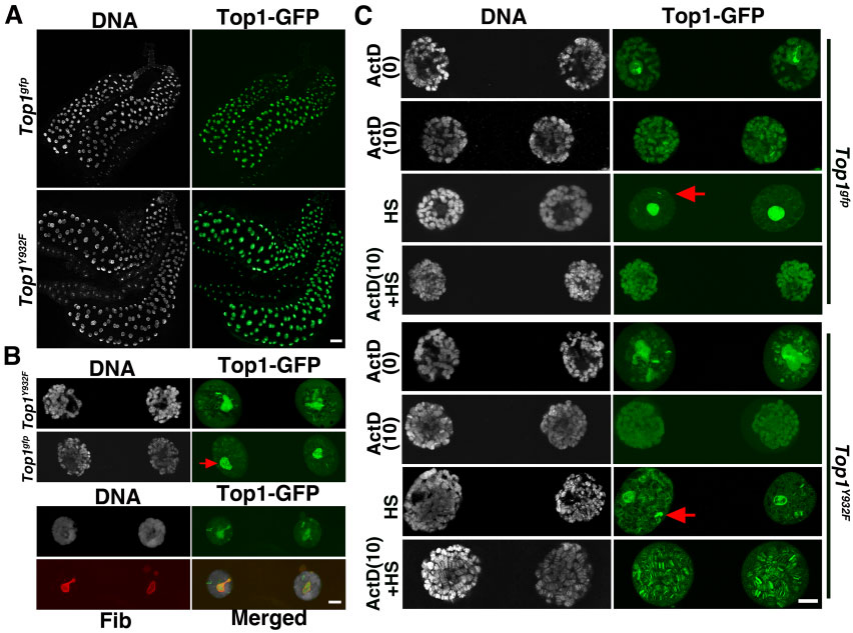
Top1-GFP localization in larval salivary glands and its regulation by transcription. (A) Top1-GFP localization in the salivary glands. Lower magnification images of salivary glands from third instar larvae expressing either Top1-GFP or Top1^Y932F^-GFP. Both proteins display strong nuclear enrichment. Scale bar represents 100µm. (B) Higher magnification images of GFP localization in salivary gland nuclei. The top panels show that Top1-GFPs are enriched at the nucleolus (indicated by an arrow). The genotypes are indicated to the left. The bottom panels show Top1-GFP and Fib colocalizing. Scale bar represents 10 µm. (C) The effect of transcriptional perturbations on Top1-GFP distribution. Both the wild type (top) and the active site mutated Top1-GFP (bottom) were tested. As the concentration of ActD increases from 0 to 10 µg/ml, Top1-GFP’s nucleolar enrichment is diminished while its chromosomal distribution seems less affected (top two rows in both panels). The middle rows show HS-treated nuclei with the position of the presumed heat shock loci indicated by arrows. The bottom rows show nuclei that were treated with ActD (10 µg/ml) followed by heat shock [ActD (10) + HS]. Scale bar represents 10 µm.

We also investigated Top1-GFP localization in another type of metabolically active cells: the giant polytene cells from the salivary glands of a third instar larva. As shown in Figure 3, A and B, Top1-GFP or its mutant form Top1^Y932F^ is highly enriched at the nucleolus but is nonuniform. Since not all *rDNA* units are transcribed (Franz and Kunz 1981; Muscarella *et al.* 1987), we suggest that the GFP-bright regions in individual nucleoli might represent *rDNA* units actively being transcribed by Pol I.

### Top1 distribution upon transcription perturbation

We applied conditions that are known to affect transcription to probe the regulation of Top1-GFP localization in larval polytene cells. First, we treated polytene cells with the known transcriptional inhibitor, ActD (Christensen *et al.* 2004). ActD is a general transcription inhibitor that affects both RNA Pol I and II polymerases. However, based on mammalian cell culture studies, the sensitivity of Pol I to ActD is one to two orders of magnitude higher than that of Pol II (Perry and Kelley 1970; Bensaude 2011). We tested a series of ActD concentrations for their effects on Top1-GFP localization and arrived at a concentration of 10 µg/ml as the lowest concentration that yields a consistent effect (Supplementary Figure S1 in Supplemental Materials). At this concentration, ActD abolished Top1-GFP localization to the nucleolus with little effect on Top1-GFP localization on chromosomes (Figure 3C). We also tested two other compounds that have been used for Pol I inhibition in mammalian cells: BMH-21 (Colis *et al.* 2014) and CX-5461 (Rossetti *et al.* 2018), even though their efficacy in transcription inhibition has not been demonstrated in Drosophila. As shown in Supplementary Figure S1, neither drug elicited a change of Top1-GFP localization at a concentration significantly higher than that used in the mammalian studies and was not pursued further. Nevertheless, our results from ActD treatment are consistent with that Top1 localization to the nucleolus depends on Pol I activities. Our results also suggest that the non-nucleolar localization of Top1 is independent of Pol I activities, but could be via other activities such as those maintained by Pol II.

We first applied the known Pol II inhibitor DRB to probe Top1-GFP localization regulation. As shown in Supplementary Figure S1, we did not observe a discernable change of Top1-GFP distribution at a DRB concentration (100 µM) similar to ones previously used (Petesch and Lis 2008; Teves and Henikoff 2011). Our second way of altering Pol II transcription was by applying a heat shock treatment (HS). HS induces a global repression of transcription that includes a reduction of paused Pol II (Teves and Henikoff 2011) and a dissociation of Pol II from chromatin in Drosophila and mammalian cells (Jamrich *et al.* 1977; Hieda *et al.* 2005). As shown in Figure 3C, a 30-min HS at 37°C resulted in a dramatic “clearing” of Top1-GFP from chromosomal sites. Our results are consistent with the scenario in which a general inhibition of Pol II transcription on genes other than the heat shock loci leads to a reduction of chromatin-bound Top1 except those at the nucleolus. We did observe a few chromosome sites with persistent Top1-GFP signals (arrows in Figure 3C) and suggest that these represent the loci encoding the heat shock proteins, which have been shown previously to accumulate Pol II and Top1 upon HS (Fleischmann *et al.* 1984; Gilmour and Elgin 1987; Shaiu and Hsieh 1998; Zobeck *et al.* 2010).

When we combined the ActD and HS treatments, we made an interesting but unexpected observation. As we showed earlier, HS leads to a dramatic clearing of Top1-GFP from chromosomal sites (the “HS” panel in Figure 3C). This clearing was halted if HS was preceded with an ActD treatment (the “ActD+HS” panel in Figure 3C), while Top1’s accumulation at the nucleolus was again greatly reduced. In essence, the effect on Top1-GFP localization from the “ActD only” treatment is very similar to that from an “ActD+HS” treatment [compare the “ActD(10)” panel with the “ActD(10)+HS” panel in Figure 3C]. It is intriguing that Top1 failed to vacate from chromatin when both rDNA transcription by Pol I and Pol II transcription genome wide were inhibited. It is possible that the ActD treatment resulted in the immobilization of Top1-GFP on chromatin as suggested by prior results in mammalian cells (Trask and Muller 1988). However, we observed similar dynamics for the active site mutated Top1-GFP (Top1^Y932F^, Figure 3C lower panels) suggesting that normal Top1 enzymatic activity was not involved in the presumed Top1 immobilization by ActD.

When we reversed the sequence of the two treatments (HS followed by ActD), neither the clearing of “chromosomal” Top1-GFP nor the accumulation of Top1-GFP in the nucleolus could be reversed by ActD (Supplementary Figure S1B). These results suggest that cells treated under the current conditions might have entered an arrested state thus are incapable of responding to further stimulations.

### Top1 enriched loci in syncytial embryos

Animals with no intact copy of the Top1 gene die as early larvae possibly due to a block of tissue proliferation (Lee *et al.* 1993; Zhang *et al.* 2000), which was similarly observed for our *Top1* alleles. Therefore, determining Top1’s function in early embryonic development requires sophisticated genetic manipulations. Based on a clever design using the heat shock promoter to drive *Top1* expression, Zhang *et al.* (2000) supplied sufficient Top1 only to somatic tissues during oogenesis so that early embryos with a reduced level of Top1 could be studied. They discovered that these Top1-insufficient embryos suffered mitotic catastrophe and arrested early in development. However, several issues remain unresolved if one considers that Top1’s primary role is in transcription regulation, in particular transcription originated from the highly active *rDNA* locus. Early cycles in syncytial embryos are run on maternal contributions without zygotic transcription. Falahati *et al.* (2016) showed that the *rDNA* locus is among one of the first zygotically active loci where rDNA transcripts can be first detected at cycle 11 and become prominent at cycle 12. Therefore, one might expect that the maternal Top1 proteins start to accumulate at *rDNA* at the time of its activation. However, results from Top1 immunostaining experiments of early embryos suggest a broad distribution in interphase nuclei and the disappearance of Top1 from mitotic chromosomes (Zhang *et al.* 2000). In addition, some of the embryos deficient for Top1 arrest earlier than cycle 12 with mitotic defects, suggesting that Top1 might be needed specifically for early cell divisions but function independently of its role in transcription since major zygotic transcription does not start until cycle 14.

**Figure 4:**
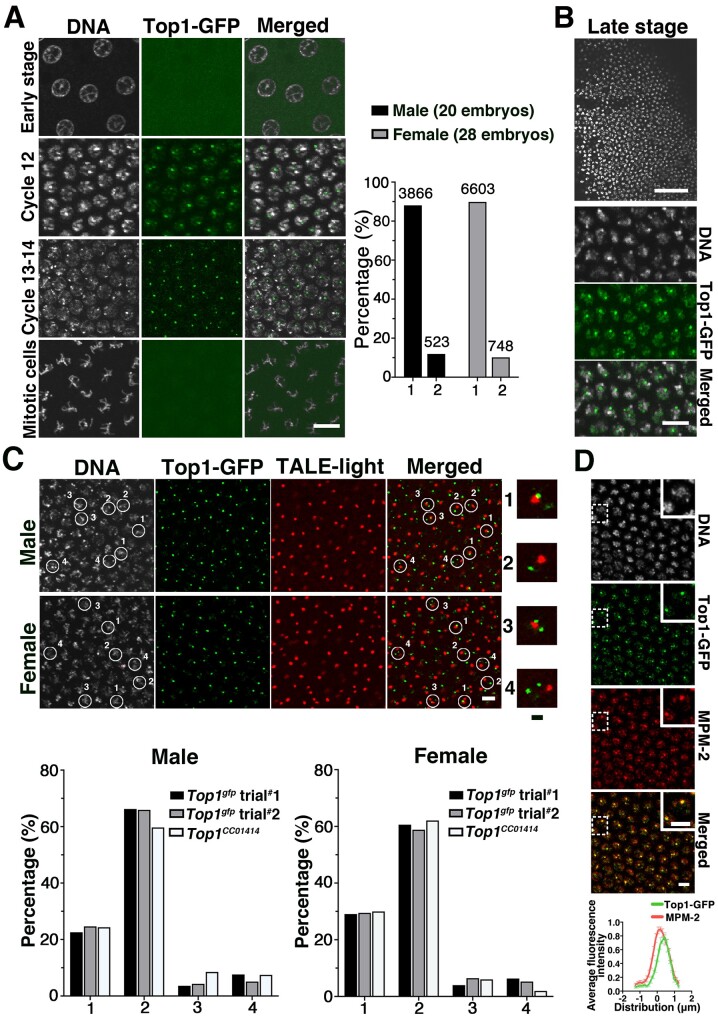
Top1-GFP localization and its relationship to *rDNA* in preblastoderm embryos. (A) The timing of Top1-GFP foci formation in syncytial embryos. An embryo at cycle 9 was used as a representative for displaying Top1-GFP localization in embryos of the “Early stage.” GFP signal was overexposed to show the lack of Top1-GFP foci in interphase nuclei. In the embryos at Cycles 12, 13, or 14, nuclear Top1-GFP foci were observed. Top1-GFP is absent from mitotic chromosomes. The quantification of the number of Top1-GFP focus per interphase nucleus is shown in the chart to the right of the images. The embryos were sexed based on the number of the *X*-linked 359 satellite locus in the embryos using the method described in Supplementary Figure S1 and (C) in this figure. Scale bar represents 10 µm. (B) Top1-GFP foci in postblastoderm embryos. At the top is a lower magnification image of an area from a postblastoderm embryo showing uneven nucleus distribution. Scale bar represents 50 µm. Higher magnification images are shown in the bottom three panels with Top1-GFP forming one to two prominent foci per nucleus. Scale bar represents 10 µm. (C) The TALE-light method for determining the positional relationship between Top1-GFP focus and the 359 satellite. The top panels show images of syncytial embryos expressing Top1-GFP (in green) that have been stained with TALE-light (in red) for labeling of the 359 satellite. The “merged” image shows GFP and TALE-light signals. Scale bar represents 5 µm. The sex of the embryos is indicated to the left of the images and was determined based on the criteria described in Supplementary Figure S1. DAPI signals were used to estimate the boundary of a nucleus, which is marked with a circle. We identified four classes of nuclei, which are labeled from 1 to 4 next to the nuclear circle. In a class “1” nucleus, the single green focus juxtaposes a red focus. In a class “2” nucleus, the single green focus does not juxtapose any of the red focus. In a class “3” nucleus, one of the two green foci juxtaposes a red focus. In a class “4” nucleus, none of the two green foci juxtaposes a red focus. Higher magnification pictures of the four classes of nuclei are shown to the right of the main image panels. Scale bar indicates 1 µm. Below the image panels is the quantification for the four classes in male (left chart) and female (right chart) embryos. (D) Top1-GFP colocalization with the MPM-2 epitope. In the merged image, only Top1-GFP and anti-MPM-2 signals are shown. *n* = 30 embryos. Magnified images of the areas marked with dashed rectangles are shown as inserts. Scale bar indicates 5 µm. A line-scan analysis from eight nuclei is provided as the bottom and error bars indicate SEM.

To gain possible insights into Top1’s role in early embryonic cycles, we studied the localization of Top1-GFP in syncytial embryos collected 0–2 h after egg laying. As shown in Figure 4A, nuclear Top1-GFP signal is undetectable in embryos cycling before cycle 12 (*n* = 7 embryos), while in those cycling at or after cycle 12 (*n* = 48) nuclear Top1-GFP is clearly visible and forms 1–2 bright foci per interphase nucleus (quantification in Figure 4A). Consistent with earlier results (Zhang *et al.* 2000), Top1 is not enriched on the mitotic chromosomes of the embryos (Figure 4A). In postblastoderm embryos, Top1 foci become more intense but again forming 1–2 foci per nucleus (Figure 4B).

The interesting coappearance of rDNA transcription and Top1-GFP foci in and around cycle 12 prompted us to investigate whether Top1-GFP foci are located at the *rDNA* loci. Drosophila has two *rDNA* gene clusters, one near the centromere of the *X* chromosome and the other on the short arm of the *Y* chromosome. Next to the *X*-linked *rDNA* is the 359 satellite that consists of about 11 Mb of middle-repetitive elements (Tang *et al.* 2017). Yuan *et al.* (2014) developed the “Tale-Light” method in which a TALE DNA-binding domain was engineered to specifically recognize a sequence in the repeated unit of the 359 satellite. When fused with the mCherry fluorescent protein and purified from bacteria, this TALE-Light protein allows visualization of the 359-satellite in fixed samples. The invention of the 359-TALE-light thus offers a unique way to approximate the localization of Top1-GFP foci in relationship with *rDNA* on the *X vs* the *Y* chromosomes since only the *X*-linked one is situated next to 359. For representative images showing the relative locations between 359 satellite and the *rDNA* locus in syncytial embryos, see Tang *et al.* (2017).

We used fixed syncytial embryos in which green fluorescence from Top1-GFP was detectable under a confocal microscope. These embryos have also been incubated with TALE-mCherry recombinant proteins similar to a primary antibody used in a traditional immunostaining experiment. When we estimated the number of TALE-mCherry foci per nucleus in a given embryo, we observed two classes that we suggest represent males and females. Representative images are shown in Supplementary Figure S2 in Supplemental Materials. In one class, presumably the female class about 10–20% of the nuclei have two TALE-mCherry foci (quantification can be found in Supplementary Table S2 in Supplemental Materials). The cases of a single focus in these embryos likely represent “pairing” of the two homologous *X*s at the 359 satellite. In the other class, presumably the male class, more than 99% of the nuclei had one TALE-mCherry focus. The rare cases of two foci in these embryos are likely the result of sister chromatid separation at the 359 regions.

As shown in Figure 4C, the majority (70–80%) of Top1-GFP foci are not situated in close proximity to a 359 satellite whether it is in a male or female embryo. This result applies to all Top1-GFP expressing stocks that we tested, and on two independent trials. Therefore, Top1-GFP focus in about 70% of the female nuclei is not at either of the *rDNA* loci on the *X*. In male embryos, on the other hand, we cannot rule out that the majority of Top1-GFP foci were actually associated with the *rDNA* locus on the *Y* chromosome therefore apart from the 359 satellite on *X*. This possibility would imply sex-specific mechanisms for Top1 recruitment so that Top1 is *rDNA*-associated in male but not female embryos. Warsinger-Pepe *et al.* (2020) reported previously that differential transcriptional activities from the *X* and *Y rDNA* loci remain insignificant in pregastrulating male embryos, similar to those that we examined. Therefore, a plausible sex-specific mechanism remains missing for localizing Top1 to the *Y*-linked *rDNA* locus but not the X-linked copy. Therefore, the most parsimonious explanation for our results would be that the single Top1-GFP focus in a male embryo is not at *rDNA*.

We therefore speculate that in a significant portion of the nuclei in preblastoderm embryos, the strongest Top1-GFP foci do not colocalize with the *rDNA* locus. Another nuclear body forms during early embryonic development is the histone locus body (HLB) that colocalizes with the *histone* gene cluster (Liu *et al.* 2006b; White *et al.* 2007). We tested the possibility that Top1-GFP accumulates at HLB by using an antibody that recognized the MPM-2 epitope, a marker for HLB in early embryos (White *et al.* 2007). Remarkably, Top1-GFP colocalizes with the strongest MPM-2 signal (Figure 4D). Although this result suggests that Top1-GFP accumulates at HLB during early development and would be consistent with Top1’s enrichment at a transcriptionally active region of the genome, the final confirmation requires colocalizing Top1-GFP with the *histone* locus and other HLB markers such as the U7 snRNP important for Histone mRNA processing (Liu *et al.* 2006b). We note that there are minor MPM-2 positive foci colocalizing with Top1-GFP signals. The molecular nature and functional significance of these secondary foci require future investigations.

### Top1-GFP may mark both *rDNA* loci in larval diploid cells

**Figure 5:**
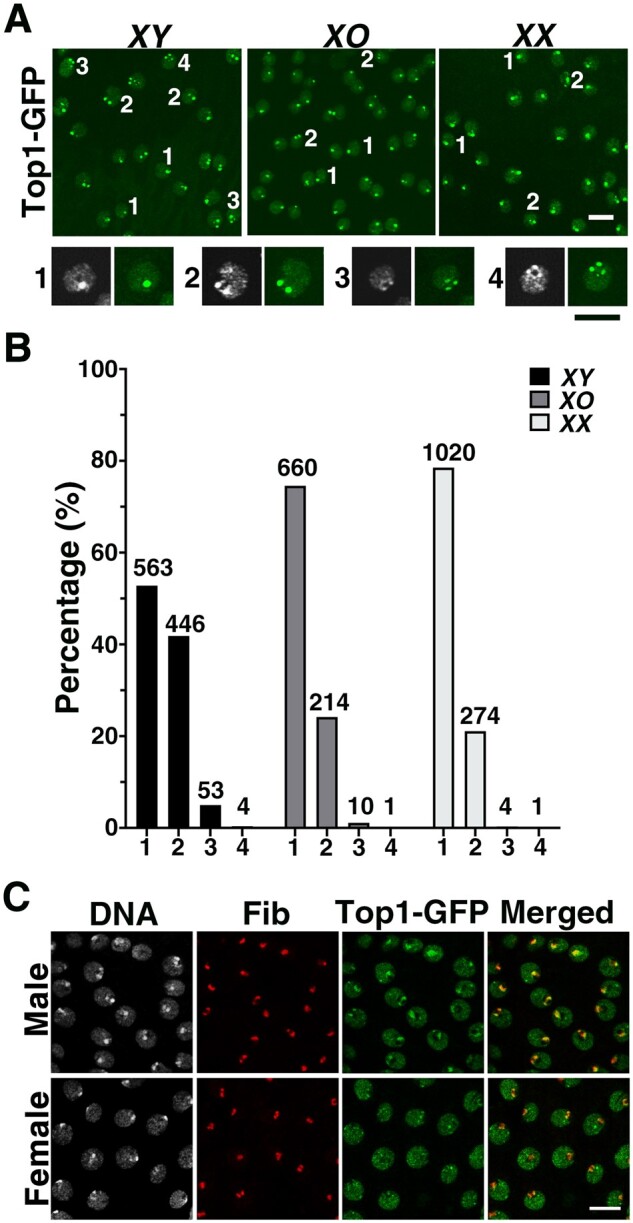
Top1-GFP focus and its relationship to the *rDNA* locus in proliferating cells of the larval wing discs. (A) Images of live wing discs expressing Top1-GFP. The number of Top1-GFP focus inside a few representative nuclei is marked with a number next to the nucleus. Higher magnification images of the four classes of nuclei are shown below the main image panels, accompanied by DNA staining of the same nuclei. Scale bars indicate 10 µm. (B) Quantification of Top1-GFP foci according to sex chromosome compositions (*XY*, *XO*, and *XX*). The classes of the nuclei are indicated on the X-axis, and the number in each class on top of the bar. (C) Top1-GFP and Fib colocalize in larval disc cells. Larval wing disc cells expressing Top1-GFP (green) were staining for DNA (white) and anti-Fib (red). The merged image only displays signals from Top1-GFP and anti-Fib. Scale bar indicates 10 µm.

We used cells in larval imaginal discs as another cell type to study Top1-GFP localization in diploid cells. In particular, these cells are primarily at interphase, different from those in early embryos that are highly active in cell divisions. We focused our live analyses on cells from the wing discs of third instar larvae. As shown in Figure 5A and quantified in Figure 5B, in 94.7–99.6% of the nuclei Top1-GFP forms 1–2 foci per nucleus. Our hypothesis was that these major foci are localized at *rDNA*. Testing of the hypothesis requires means to simultaneously identify *rDNA* and Top1-GFP loci in disc cells.

When we performed immunostaining with anti-Fib in these cells, the Top1-GFP signal overlaps with that of Fib antibody suggesting that at least some of the Top1-GFP is at the nucleolus (Figure 5C). However, these results from immunostaining were unsatisfactory as GFP signals are too diffuse to define as clear a focus as we were able to do in live analyses. Moreover, although Fib is a common marker for the nucleolus, which is derived from active rDNA transcription, Fib could not indicate the location of the inactive *rDNA* locus, which is important to identify in males as discussed later. In addition, we attempted but unsuccessfully to apply the TALE-light method described before in larval imaginal discs. Without the ability to simultaneously identify both *rDNA* and Top1-GFP loci in discs, we nevertheless investigated the distribution pattern of Top1-GFP focus number using larvae carrying different combinations of the sex chromosomes: *XX*, *XY*, and *XO* to extract any relationship between Top1-GFP and *rDNA*, but without the a priori assumption that Top1-GFP is located at the *rDNA* loci.

As third instar larvae can be sexed visually (Kerkis 1931), we first investigated whether the number of Top1-GFP focus correlates with the sex of the animal. As shown in Figure 5B, we observed a significant increase of nuclei with two foci in male *vs* female larvae (*P *<* *0.000001 from a 2X2 contingency test). Since the only difference in chromosome composition between the two sexes lies in the sex chromosomes, the change in Top1-GFP focus number was likely brought about by the same mechanism, suggesting that Top1-GFP foci are likely on one of the two sex chromosomes. In addition, the increase of the two-focus class in males is inconsistent with that all of the Top1-GFP foci are on the *X* chromosome since males have one less *X* than females. Therefore, at least some of the foci must be on the *Y* chromosome. This would be consistent with Top1-GFP foci at *rDNA* as it is a homologous locus between the sex chromosomes.

Although a female nucleus has two *rDNA* loci on each of its two *X*s, the somatic pairing would reduce the average closer to one when observed cytologically. As the *X* and the *Y* are not homologous and hence not paired, a male nucleus with two foci might represent Top1-GFP accumulation on both of its sex chromosomes. To provide additional support for this hypothesis, we measured the number of Top1-GFP focus in wing disc cells from *XO* larvae. Again, we observed predominantly one or two foci per nucleus (Figure 5B). The distributions of nucleus between one and two foci are not statistically different between *XO* and *XX* discs (*P *=* *0.07), consistent with our suggestion that the number of *rDNA* locus in a female nucleus is effectively one due to somatic pairing. The two-foci class in an *XO* nucleus could be the result of transient sister chromatid separation at *rDNA*. Interestingly, the portion of *XY* nuclei with two Top1-GFP foci is significantly greater than that of *XO* nuclei (*P *<* *0.000001). Since this increase must have been brought about by the presence of the *Y* chromosome, we suggest that at least in some of these two-foci nuclei both *rDNA* loci were marked with Top1-GFP accumulation.

An important alternative explanation of the above results is that one or both of the Top1-GFP foci are located at places unrelated to *rDNA*, such as the HLB similar to what we observed in embryos (Figure 4D). However, this model would have to correlate the number of Top1-GFP focus with changes in the number (one or two) and composition (*X* or *Y*) of the sex chromosomes, and we find that difficult since the *histone* cluster is on chromosome *2*.

Recently, the phenomenon of “nucleolus dominance” was described in *Drosophila melanogaster* in that somatic cells of males express rRNA from the *Y* chromosome, while the *rDNA* locus on the *X* is silenced (Greil and Ahmad 2012; Zhou *et al.* 2012; Warsinger-Pepe *et al.* 2020). As Top1 recruitment to chromatin has been frequently linked with transcription, we suggest that in an *XY* nucleus with a single Top1-GFP focus, it is likely associated with the active *Y rDNA*, while a two-foci nucleus has both *rDNA* loci marked with Top1. Our data thus suggest that *rDNA* on the *X* chromosome in males is also capable of attracting Top1 even when it is transcriptionally silenced. It is possible that a transcription-independent recruitment mechanism exists for Top1 as what has been reported in Tetrahymena in which a sequence fragment contained in the intergenic sequence (IGS) of Tetrahymena rDNA displays a high affinity for Top1-binding *in vitro* (Bonven *et al.* 1985). A highly homologous sequence also exists in the IGS of Drosophila rDNA. Unfortunately, we failed repeatedly to introduce a DNA fragment containing 32 copies of the 240-bp IGS into the Drosophila genome. This precluded us from testing the hypothesis that sequence-specific binding of Top1 to IGS serves as another mechanism for Top1 recruitment in Drosophila. We note that Thomas and McKee (2007) succeeded in introducing an eight-copy IGS fragment carried on a transgene.

### Top1 marks the *X-Y* bivalent in meiotic cells and potentially function to ensure *X-Y* disjunction

Top1’s function in transcription regulation has been extensively studied during mitotic growth. Its meiotic function is less clear. We set out to investigate Top1-GFP distribution in testicular cells out of consideration of a special relationship between *rDNA* and meiotic chromosome segregation in Drosophila males [for a review of Drosophila male meiosis, see McKee *et al.* (2012)]. The landmark study by McKee and Karpen (1990) established that the *rDNA* loci on the sex chromosomes mediate their conjunction to ensure their segregation during meiosis I. It was subsequently proposed by McKee *et al.* (1992) that Top1, known to be enriched at the *rDNA* loci in somatic cells, might serve to regulate *X-Y* disjunction in Drosophila. In addition, the Top1-interacting dTopors protein regulates chromosome segregation in Drosophila males (Matsui *et al.* 2011).

**Figure 6:**
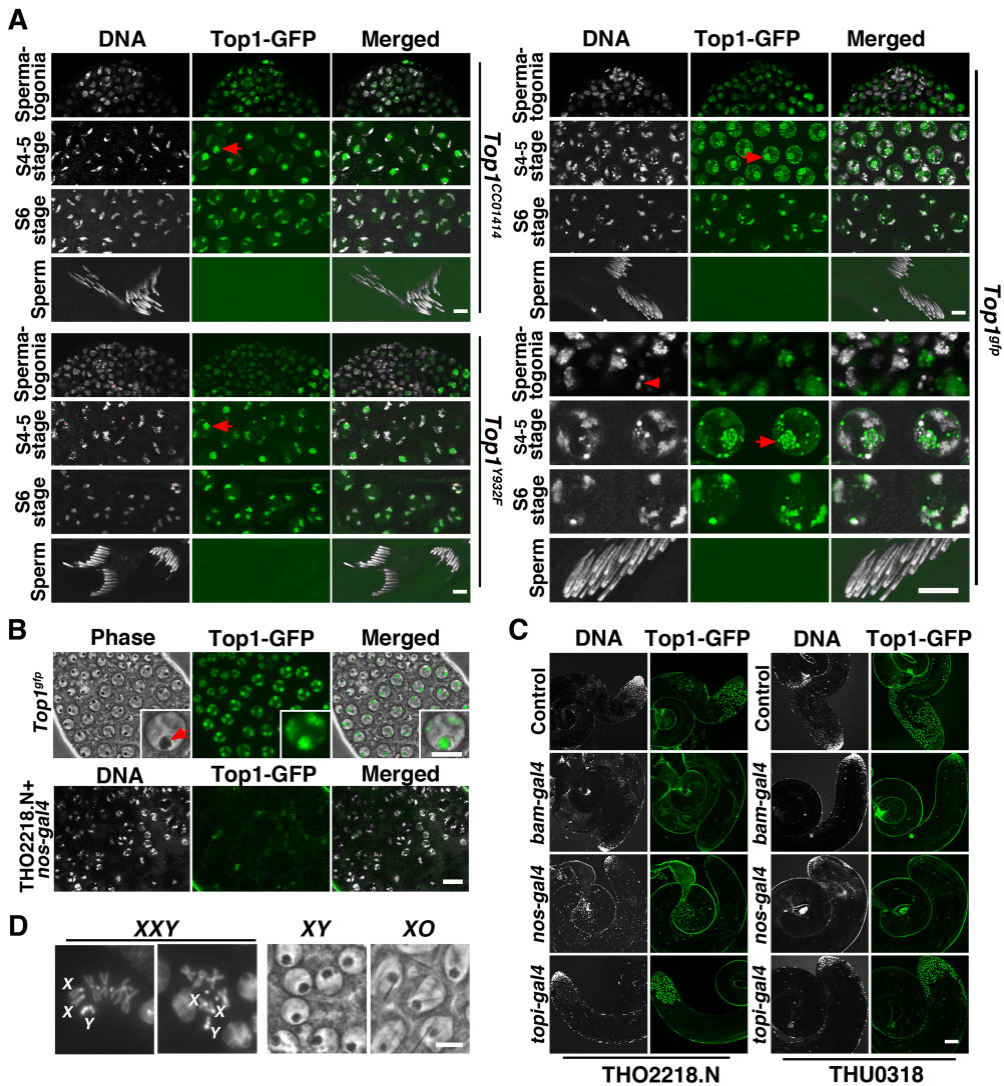
Top1-GFP localization in the testis. (A) Top1-GFP distribution in different stages of spermatogenesis. The stages are indicated to the left. For a detailed classification of stages during spermatogenesis, see Cenci *et al.* (1994). Three alleles of Top1-GFP are used with the genotypes indicated to the right of the image panels. The potential *rDNA* foci are indicated by arrows. For the *Top1^gfp^* allele, magnified images are also provided and organized similarly according to stages of development. In the “Spermatogonia” panel, a mitotic nucleus is marked with an arrowhead. Scale bar represents 20 µm. (B) Top1-GFP KD in primary spermatocytes. Images from phase-contrast microscopy (Phase) and live fluorescent microscopy (GFP) are shown for primary spermatocytes from a wild-type male (top) expressing Top1-GFP. Magnified images of a single spermatocyte nucleus are shown as inserts. The position of the nucleolus is indicated by an arrow. Images from DAPI staining and live fluorescent microscopy (GFP) are shown for a male experiencing Top1-GFP KD driven by nos-Gal4 (bottom). Scale bar indicates 10 µm. (C) Top1-GFP KD by different Gal4 drivers. Lower magnification images of “control” and KD testes are shown to indicate the extent of Top1-GFP reduction. The images are grouped based on the Gal4 drivers (shown to the left) and RNAi lines (shown at the bottom). Scale bar indicates 100 µm. (D) Validation of exceptional progeny from NDJ testcrosses. The top two images show the presence of the extra *Y* chromosome from an *XXY* nucleus with all the sex chromosomes labeled. The bottom two images are primary spermatocytes from an *XY* (top) and an *XO* (bottom) male showing the presence of needle shape crystals in *XO* spermatocytes. Scale bar indicates 10 µm.

As shown in Figure 6A, Top1-GFP forms foci in cells from the mitotic compartment of the testis. In particular, Top1-GFP foci are the largest in size in spermatocytes (Figure 6, A and B), consistent with the high transcriptional activity in these cells. Using phase-contrast microscopy, we observed colocalization between the strongest Top1-GFP foci and the nucleolus in these cells (Figure 6B). These Top1-GFP foci juxtapose the *X-Y* bivalent when they become visible after the initiation of meiotic chromosome condensation (“S4-5 stage” in Figure 6A). Interestingly, even in cells with highly condensed meiotic chromosomes, Top1-GFP are prominently located on regions of chromosomes (“S6 stage” in Figure 6A).

To test whether Top1 participates in regulating *X-Y* disjunction, we used RNAi to reduce Top1 level in the testes as *Top1* knockout mutations cause lethality. As shown in Figure 6, B and C, our Top1-GFP can be effectively knocked down (KD) by RNAi reagents driven by the germline-specific *bam-gal4* or *nos-gal4* drivers. Therefore, using Top1-GFP as a reporter, we demonstrated that Top1 could be effectively KD, at least in the mitotic compartment of the testis.

Using a marked *Y* chromosome (*B^S^Yy^+^*), we investigated whether Top1 KD in the male germline induces *X-Y* nondisjunction (NDJ). Normally, the *Y* chromosome from the father is transmitted solely to its male progeny. In the event of an *X-Y* NDJ, progenies of two exceptional classes are recovered: sons bearing no *Y* chromosome (*XO*) and daughters bearing a *Y* chromosome (*XXY*). As shown in Table 1, when Top1 was KD, *X-Y* NDJ at a rate about 10-fold higher than the normal rate was indeed observed. To confirm that the exceptional classes of progenies indeed carry the expected sex chromosome compositions, we performed additional tests as followed. First, all suspected *XO* sons were confirmed to be sterile by test-mating with wild-type females, and they were subsequently dissected to reveal the presence of needle-shaped crystalline in primary spermatocytes (Figure 6D), a cellular phenotype consistent with the absence of the *Y* chromosome (Hardy *et al.* 1981). Secondly, some of the *XXY* offspring were mated to wild-type males and some of their progenies were subjected to karyotyping in which neuroblasts from female third instar larvae were subjected to a mitotic chromosome squash assay. Some of the squashes clearly display the presence of the extra *Y* chromosome (Figure 6D). The presence of the extra *Y* in any of the daughters of a suspected F1 female confirms that this exceptional female in question was indeed *XXY*. Importantly, it is the presence of these *XXY* exceptional progenies that indicates that these exceptional events were the result of meiosis I NDJ.

**Table 1:** *X*-*Y* NDJ frequencies in Top1 KD males

	*XX*	*XY*	*XO*	*XXY*	Total	NDJ%	N
*yw/B^S^Yy^+^;THO2218.N/+*	2,129	1,392	0	0	3,521	0	31
*yw/B^S^Yy^+^;THO2218.N/+; bam-gal4/+*	456	314	0	0	770	0	12
*yw/B^S^Yy^+^;THO2218.N/+; topi-gal4/+*	1,112	1,015	4	4	2,135	0.37*a*	28
*yw/B^S^Yy^+^;THU0318/+*	1,929	1,274	1	0	3,204	0.03	31
*yw/B^S^Yy^+^;THU0318/bam-gal4*	484	335	0	0	819	0	11
*yw/B^S^Yy^+^;THU0318/nos-gal4*	1,113	620	4	2	1,739	0.35*a*	21
*yw/B^S^Yy^+^;THU0318/topi-gal4*	907	851	4	1	1,763	0.28*b*	22

The genotypes of the test males are shown in the leftmost column. THO2218.N and THU0318 are two independent *Top1* RNAi lines. The four classes of progeny were scored for each testcross according to their sex chromosome compositions. The frequency of nondisjunction (“NDJ%”) was calculated by dividing the sum of exceptional progenies (*XO* and *XXY*) by the total number of progenies. “N” indicates the number of fertile male parents tested for NDJ.

a A 2 × 2 contingency test with the non-RNAi control yield *P* < 0.01.

b A 2 × 2 contingency test with the non-RNAi control yield *P* < 0.05.

One possible cause for the appearance of the above-described exceptional offspring is mitotic NDJ event, involving sister chromatids for example, which could be brought about by chromosome exchanges between the *rDNA* loci. This would result in germ cells that are *XO*, *XXY*, *XYY*, or *XXYY*. We suggest this to be unlikely based on the following considerations. First, an *XO* spermatogonium would be nonunctional due to the absence of the *Y*-linked fertility factors. It is therefore difficult to account for the *XO* exceptional male offspring based on mitotic NDJ alone. Secondly, the exceptional progenies were all from different fathers inconsistent with the expected “clustering” of mitotic events. The caveat here is that the number of exceptional offspring was much smaller than the number of male parents tested in our experiments. Lastly, a physical linkage due to an aberrant exchange between the *rDNA* loci on the *X* and *Y* could have forced them to mis-segregate in meiosis I. We suggest that this would be resolved in the offspring leading to the formation of *X*- and *Y*-derived chromosomes, similar to those derived from *rDNA* exchanges induced by the I-CreI endonuclease (Maggert and Golic 2005). Our sampling of the *XXY* females by karyotyping their offspring offers no support for the existence of such aberrant chromosomes (Figure 6D).

Data shown in Table 1 appear to suggest that not all *gal4* drivers were equally effective in inducing *X-Y* NDJ. However, although differences in NDJ frequency between Top1 KD germlines and the non-RNAi controls reach statistical significance except for the cases of *bam*-driven KD, differences between *bam*-driven KD *vs nos*-driven or *topi*-driven KD are not statistically significant (*P* = 0.12 for the THO2218.N siRNA and *P* = 0.19 for THU0318). We nevertheless speculate that different stages of Gal4 expression might affect meiotic chromosome segregation to different degrees. The expression of the *bam* gene is limited to the mitotically amplifying cells in the spermatogonium (Schulz *et al.* 2004). Importantly, *bam* expression is very low or absent in spermatocytes right before meiosis. Consistently, we observed normal *X-Y* disjunction in *bam-gal4* driven RNAi KD germline. Importantly, many of these males were sterile or semisterile, consistent with that Top1’s reduction in mitotic cells likely causes germ cell loss. Interestingly, the *nos-gal4* has a wider expression than *bam* including detectable expression in spermatocytes (Schulz *et al.* 2004). Consistently, the *nos-gal4* driven Top1 KD induced NDJ at a significant rate. These males also suffer reduced fertility possibly owning to Top1 knockdown in proliferating cells. *topi-gal4* has a narrower window (just prior to meiosis) and lower level of expression than either *bam-gal4* or *nos-gal4* (Raychaudhuri *et al.* 2012). Consistently, it is not very efficient in directing Top1 KD in the premeiotic compartment (Figure 6C), and the “KD” males had normal fertility. Remarkably, topi-Gal4 directed Top1 KD is as effective in inducing *X-Y* NDJ as the more potent nos-Gal4, consistent with our proposition that the timing of Top1 KD is important in inducing NDJ.

Although our RNAi-mediated Top1 knockdown resulted in an *X-Y* NDJ rate (0.3%) significantly above the baseline level, it remains two orders of magnitude lower than that observed for male hemizygous for the *rDNA* locus (essentially random *X-Y* segregation, hence 50%). Other known mutants affecting *X-Y* segregation have a range of NDJ of 20–50% (*e.g.*, Thomas *et al.* 2005; Matsui *et al.* 2011), again significantly higher than what we observed here. This could be due to that Top1 is not critically involved in directing *X-Y* disjunction. Alternatively, the small effect of Top1 KD on *X-Y* disjunction could be due to the ineffectiveness of our current system in reducing Top1 level.

The potential role of Top1 in mediating meiotic chromosome segregation, as proposed by McKee *et al.* (1992), was not meant to be limited to the sex bivalent, but rather applicable to chromosomes undergoing achiasmatic pairing. In our analyses, Top1-GFP signals are clearly present on autosomes (“S6 stage” in Figure 6A), consistent with a boarder role of Top1 proposed earlier. A well-timed elimination of Top1 molecules in premeiotic cells might nevertheless be essential to efficiently impair chromosome conjunction in meiosis. We plan to continue developing tools to test this hypothesis. Until then we suggest that it remains possible that Top1 is required for meiotic chromosome disjunction in Drosophila males.

### Concluding remarks

In this study, we conducted a comprehensive characterization of the localization of the important chromosomal protein Top1 during the complex developmental program of a metazoan. Our results confirm a prominent enrichment of Top1 at the *rDNA* locus and its derived structure, the nucleolus. In addition, we uncovered Top1 localization seemingly unrelated to ongoing rDNA transcription particularly in diploid cells, such as Top1 foci not situated at *rDNA* in preblastoderm embryos, Top1 foci on both the active (*Y*-linked) and inactive (*X*-linked) *rDNA* loci in cells from larval wing discs, and Top1 foci in transcriptionally quiescent meiotic cells in the testis. These results suggest the existence of additional Top1 recruitment mechanisms that might be related to its function in DNA metabolism.

## Supplementary Material

jkab202_Supplementary_Data
