# Supplementary material for: Dynamic localization of DNA topoisomerase I and its functional relevance during Drosophila development
Source: G3 (Bethesda). 2021 Jun 14;11(9):jkab202. doi: 10.1093/g3journal/jkab202 (PMC8661406; doi:10.1093/g3journal/jkab202)
Supplement: jkab202_Supplementary_Data [file jkab202_supplementary_data.docx]

**Supplemental Materials for Huang et al. 2021**

**Table S1. *Top1^gfp^* rescues the lethality of a *Top1* frameshift mutation**

| father | *w/Y; [w^+^, Top1^gfp^]/+* | | | |
| --- | --- | --- | --- | --- |
| mother | *w Top1^40^/FM7a, w* | | | |
|  | son | | daughter | |
| sperm  egg | *Top1^gfp^* | *+*  (no transgene) | *Top1^gfp^* | *+*  (no transgene) |
| *Top1^40^* | 416 | 0 | 304 | 329 |
| *Top1^+^* | 98 | 129 | 218 | 260 |

Parental genotypes are listed at the top. About 20 pairs were mated. Only the relevant genotypes are listed for the sperm and egg.

**Figure S1. The effect of RNA polymerase I and II inhibitors on Top1-GFP distribution.**


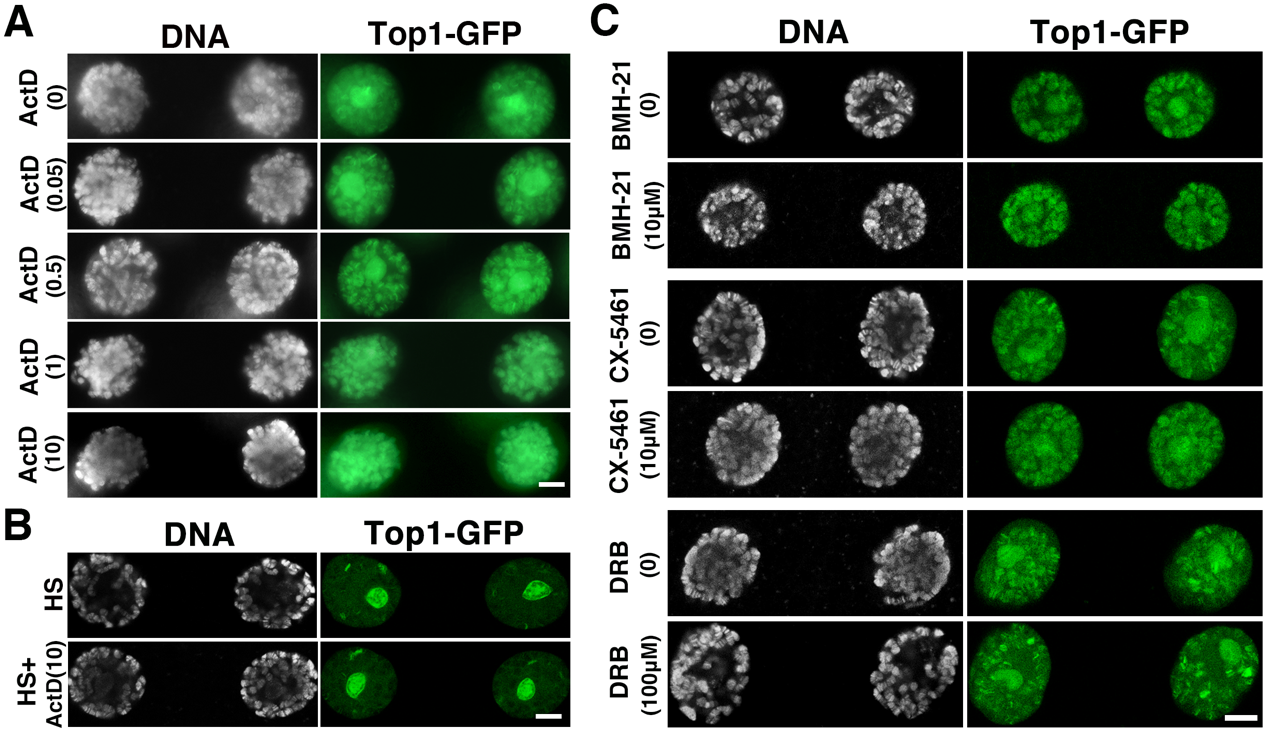


**A**. Actinomycin D treatment. As the concentration of Actinomycin D (ActD) increases from 0 to 10μg/ml, Top1-GFP’s nucleolar enrichment is diminished while its chromosomal distribution seems less affected. **B**. HS and ActD combined treatments. Salivary glands were treated with a heat shock only (HS) or with a heat shock followed by ActD (HS+ActD(10)). **C**. Other inhibitors. BMH-21 and CX-5461 are RNA polymerase I inhibitors, and DRB is a reversible inhibitor of RNA polymerase II. Salivary glands were treated in these drugs for 3h, in a concentration indicated in the parenthesis. Scale bar represents 10μm.

**Figure S2. Embryo sexing by the number of TALE-light focus.**


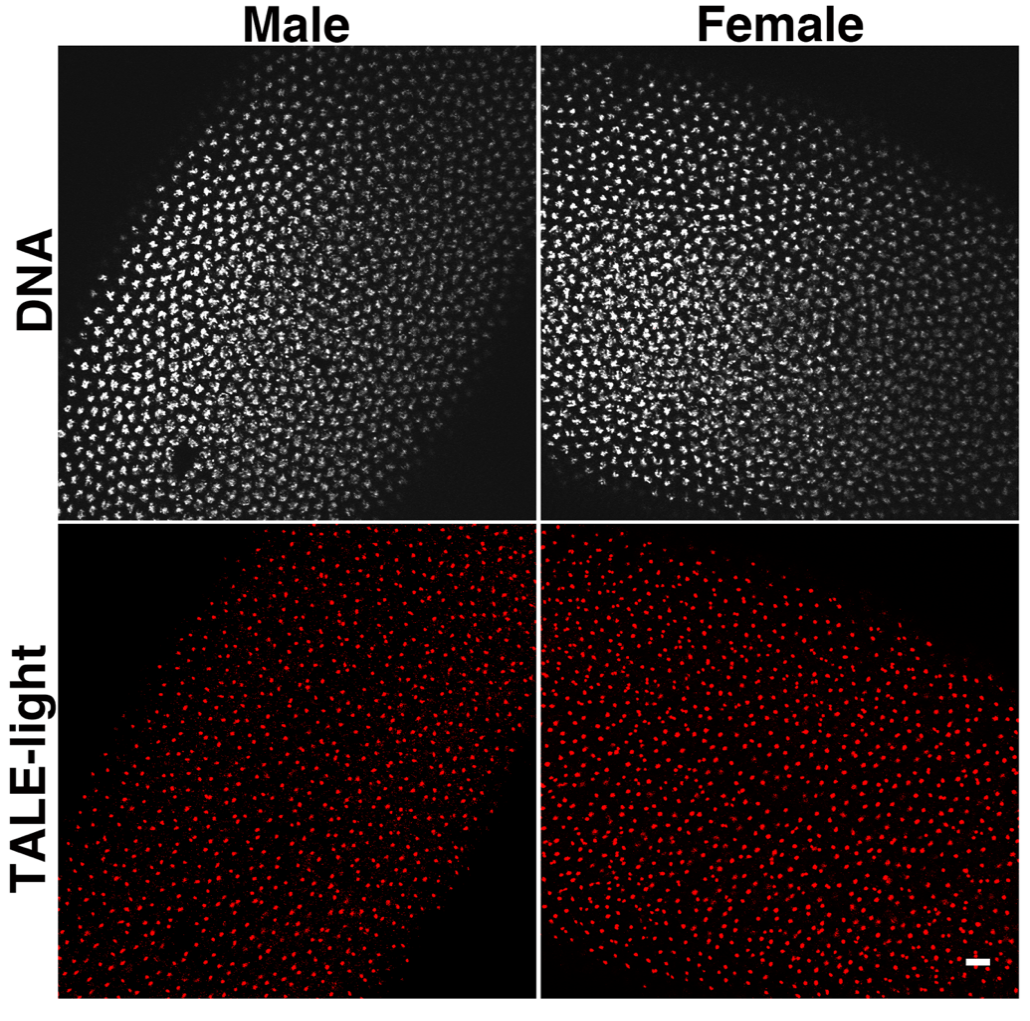


Fixed embryos stained with both DAPI and TALE-light are shown. The numbers of embryos and nuclei scored are given in the Table S1 below. Scale bar represents 10μm.

**Table S2. The number of TALE-light focus in embryos.**

| Embryo ^1^ | 1 focus ^2^ | 2 foci ^2^ | % for 2 foci | Classification ^3^ |
| --- | --- | --- | --- | --- |
| **No.1** | 601 | 0 | 0 | Male |
| **No.2** | 682 | 0 | 0 | Male |
| **No.3** | 713 | 0 | 0 | Male |
| **No.4** | 829 | 0 | 0 | Male |
| **No.5** | 557 | 1 | 0.2% | Male |
| **No.6** | 769 | 2 | 0.3% | Male |
| **No.7** | 343 | 3 | 0.9% | Male |
|  |  |  |  |  |
| **No.8** | 239 | 34 | 12.5% | Female |
| **No.9** | 350 | 73 | 17.3% | Female |
| **No.10** | 362 | 80 | 18.1% | Female |
| **No.11** | 389 | 90 | 18.8% | Female |
| **No.12** | 457 | 110 | 19.4% | Female |
| **No.13** | 478 | 118 | 19.8% | Female |
| **No.14** | 238 | 67 | 22.0% | Female |
| **No.15** | 580 | 170 | 22.7% | Female |

^1^: A total of 15 embryos were scored for TALE-light focus number.

^2^: the number of nuclei with 1 or 2 foci.

^3^: The sex of the embryos was classified based on the portion of nuclei with 2 foci: male being less than 1% and female being greater than 10%.
